# Myiasis infestation in advanced lip squamous cell carcinoma due to COVID-19 pandemic-related treatment delays

**DOI:** 10.1590/S1678-9946202567006

**Published:** 2025-02-07

**Authors:** Lucas Emanuel Macena da Silva, Natália Vitória de Araújo Lopes, Diego de Sena Costa de Oliveira, Luiz Eduardo Marinho Vieira, Hianne Cristinne de Morais Medeiros, Bruno Augusto Benevenuto de Andrade, Carolina Carvalho de Souza, John Lennon Silva Cunha, Luan Éverton Galdino Barnabé

**Affiliations:** 1 Centro Universitário de Patos, Departamento de Odontologia, Campina Grande, Paraíba, Brazil; 2 Universidade Estadual da Paraíba, Departamento de Odontologia, Programa de Pós-Graduação em Odontologia, Campina Grande, Paraíba, Brazil; 3 Universidade Federal do Oeste da Bahia, Centro de Ciências Biológicas e da Saúde, Barreiras, Bahia, Brazil; 4 Universidade Federal do Rio de Janeiro, Faculdade de Odontologia, Departamento de Patologia e Diagnóstico Oral, Rio de Janeiro, Rio de Janeiro, Brazil

**Keywords:** Myiasis, Head and neck squamous cell carcinoma, Cancer

## Abstract

Myiasis, a condition stemming from the parasitic infestation of Diptera fly larvae, constitutes a complication for cancer patients, particularly those with malignant skin wounds. The factors that contribute to myiasis include old age, inadequate hygiene, poor living conditions, vascular disease, and diabetes. Cases of myiasis in neoplastic wounds in the head and neck region are rare and guidelines or recommendations regarding the best treatment approach remain lacking. Herein, we describe a case of myiasis that developed into an extensive squamous cell carcinoma of the lip in an older male adult due to the delay in oncological treatment stemming from the COVID-19 pandemic. Patients with oral squamous cell carcinoma, especially those residing in rural areas, face a notable risk of developing oral myiasis. Therefore, it is imperative that patients and caregivers adopt strict preventive measures to avoid fly infestations in wounds. Maintaining optimal hygiene (including meticulous cleaning with antiseptic solutions before daily dressing changes) is essential to prevent myiasis. Adequate wound coverage is crucial, especially during warmer seasons.

## INTRODUCTION

Head and neck squamous cell carcinoma (HNSCC) constitutes a significant global health issue due to its high mortality, recurrence, and metastasis rates^1,2^. Patients with advanced-stage oral squamous cell carcinoma (SCC) show several signs and symptoms—such as pain, fatigue, weight loss, airway obstruction, voice changes, bleeding, tissue necrosis, and facial disfigurements—that negatively affect their quality of life.^2^ In advanced stages, these lesions, which are often incurable, can become infested by fly larvae due to continuous exposure and lack of adequate hygiene^3^.

Myiasis, a rare clinical condition due to the invasion of body tissues by dipteran fly larvae, is considered an endemic dermatosis in tropical and subtropical countries, occurring more frequently in patients with psychiatric or immune system disorders, older adults, and those living in rural areas^3^. The association between myiasis and HNSCC is uncommon, with only a few cases reported in the literature^3-20^. Infestation mainly occurs in terminally ill patients as malignant wounds attract flies due to the unpleasant odor or secretions from ulcerating tumors^3^.

The COVID-19 pandemic has exacerbated challenges in diagnosing and treating HNSCC. Social isolation measures to contain the transmission of the virus hampered access to early diagnosis and adequate treatment, resulting in critical delays and negative impacts on patients’ prognoses. We report a case of advanced-stage oral SCC in an older patient that then worsened by a myiasis larvae infestation due to delay in treatment due to COVID-19 restrictive measures. Furthermore, we highlight the complexities and challenges due to the pandemic in managing serious medical conditions (such as oral cancer) and emphasize the importance of an integrated approach that encompasses effective prevention and treatment.

## CASE REPORT

**Figure 1:**
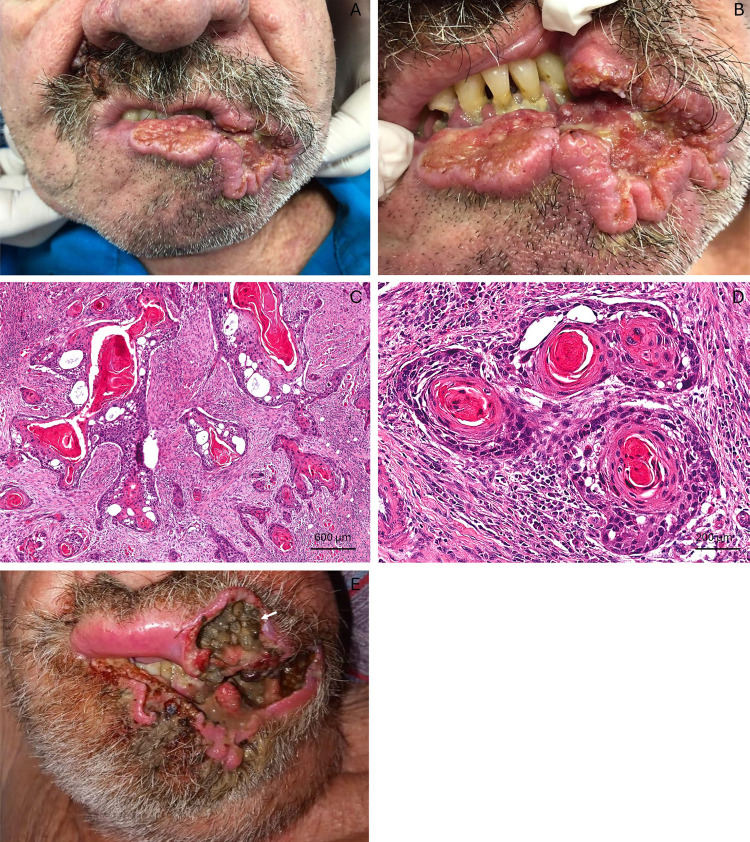
(A, B) Initial clinical presentation of the patient, showing an extensive ulcerated lesion with raised edges in the lower lip region involving the adjacent skin; (C, D) Histopathological microphotographs of squamous cell carcinoma of the lip, showing tumor islands with atypical keratinocytes and inner keratin pearls, accompanied by a discrete inflammatory predominantly lymphocytic infiltrate around the tumor islands; (E) Observation of the patient’s lesion two months after the cancer diagnosis, highlighting the infestation by numerous larvae (white arrow).

A 69-year-old White Brazilian male living in the rural area of Campina Grande, in Northeastern Brazil, who required a wheelchair to move was taken to a dental office by his niece, complaining of difficulty eating and drinking fluids. During anamnesis, the patient reported being a former smoker, having smoked 40 cigarettes a day for 35 years, and disclosed that he had consumed alcohol daily until the moment of the consultation. The patient had type 2 diabetes mellitus and his glycemic level equaled 260 mg/dL, having neither medication nor nutritional control. The extraoral physical examination observed an extensive ulcerated lesion with irregular limits and raised edges in the left lip commissure that extended into the skin. The lesion, measuring approximately 10 cm, was reddish in color, had necrotic areas, and had been there for about three months (Figures 1A and 1B). Palpation of the lymph nodes showed a firm, fixed, and painless nodule in the left submandibular region that was compatible with a metastatic lymph node.

The patient had limited mouth opening due to the lesion location, and advanced periodontal disease had compromised oral health. Clinical assessment was further complicated by the patient’s psycho-emotional state as he expressed fear of dentistry. The severity of the lesion and suspicion of malignancy entailed an incisional biopsy, and the sample was sent for histopathological evaluation. Histopathological evaluation confirmed the diagnosis of well-differentiated SCC (Figures 1C and 1D). The patient was referred to an oncology reference hospital but, due to the high demand from the COVID-19 pandemic, he unfortunately waited for four months and sadly died before starting treatment.

While waiting for treatment, approximately two months after the cancer diagnosis, our team was contacted via teleconsultation to evaluate a possible infection by fly larvae. The lesion was infested by several larvae (Figure 1E). The patient was referred to an emergency service to remove the larvae, debride the necrotic tissue, and initiate a triple therapy regimen consisting of ivermectin, albendazole, and clindamycin. The larvae belonged to the *Lucilia sericata* species (Diptera: Calliphoridae) by morphological examination. Despite the success of myiasis treatment, the patient died two months later without starting oncological treatment.

## DISCUSSION

The literature reports few well-documented cases of myiasis in patients with HNSCC, most of which occurs in Brazil and India^3-20^. Despite the limited literature, myiasis infestations in head and neck cancer lesions may occur more often than reported. This is partly due to many patients with HNSCC have ulcerative and necrotic lesions in advanced stages, which tend to be neglected and exposed to the environment, creating an environment that ripe for fly larvae infestation^3^. Furthermore, many such patients live in rural areas with inadequate infrastructure and poor hygiene habits, which increase the risk of myiasis^3^. The situation is worsened by the fact that most of these patients undergo intensive treatments, such as surgery, radiotherapy, and chemotherapy, which can complicate wound healing and worsen pre-existing malnutrition^3^.

In this case, the patient was an alcoholic, chronic smoker, and decompensated diabetic older adult with a low educational level diagnosed with advanced SCC, reinforcing these observations. Despite immediate referral to an oncology service, treatment onset was delayed due to the high demand for health services during the COVID-19 pandemic, which further compromised the prognosis. Lack of hygiene and socioemotional conditions led to a secondary larval infection. During the COVID-19 isolation period, the patient’s family contacted the teledentistry service for assistance in managing myiasis. The patient was subsequently taken to an emergency department, which removed the larvae. However, two months later, the patient died without having started cancer treatment.

In addition to the physical challenges^3,8,19^, myiasis in patients with oral SCC also has significant emotional implications^3^. Larvae cause profound emotional and psychological discomfort^3^, exacerbating the suffering associated with a cancer diagnosis, increasing anxiety and emotional distress, and decreasing patients’ ability to face the disease and adhere to treatment. This highlights the importance of not only appropriate treatment of myiasis but also of comprehensive psychosocial support to promote the holistic well-being of patients throughout the course of the disease.

Myiasis management generally requires the mechanical removal of the larvae, often combined with topical and/or systemic therapy to force migration of the larvae to the tissue surface, facilitating their removal^3,20^. Several chemical substances serve this purpose, such as ether, 70% alcohol, chloroform, turpentine, and hydrogen peroxide.^20^ Although mechanical removal is essential, systemic therapy with anthelmintics, such as ivermectin, has also been effective in eliminating larvae^3,20^. A more recent therapeutic approach involves a triple therapy with ivermectin, albendazole, and clindamycin and dressings containing turpentine oil, which has been shown to effectively eliminate the worms in three days, relieving associated symptoms and improving the general well-being of patients^20^. However, more research is needed regarding the effectiveness of these treatments and the development of new preventive strategies. Tetanus prophylaxis is also recommended to reduce the risk of tetanus infection in cases of myiasis.

## CONCLUSION

In summary, patients with head and neck SCC, especially those in poorer circumstances, show increased risk of developing myiasis. Therefore, it is essential to adopt comprehensive preventive approaches, including basic health care, hygiene promotion, fly population control, and community education on proper wound prevention and treatment to avoid new cases of maggot infestation and associated complications. The COVID-19 pandemic has further highlighted the need for adaptive and collaborative strategies to address emerging challenges in healthcare delivery.
